# The challenges and opportunities of scRNA-seq in COVID-19 research and clinical translation

**DOI:** 10.1186/s12985-022-01923-x

**Published:** 2022-12-15

**Authors:** Wendao Liu, Xiaohua Ye, Zhiqiang An, Zhongming Zhao

**Affiliations:** 1 Center for Precision Health, School of Biomedical Informatics, The University of Texas Health Science Center at Houston, 7000 Fannin St. Suite 600, Houston, TX 77030 USA; 2 MD Anderson Cancer Center UTHealth Graduate School of Biomedical Sciences, Houston, TX 77030 USA; 3 Texas Therapeutics Institute, Brown Foundation Institute of Molecular Medicine, The University of Texas Health Science Center at Houston, Houston, TX 77030 USA

**Keywords:** COVID-19, SARS-CoV-2, Single-cell RNA sequencing, COVID-19 sequela, Single-cell VDJ sequencing

## Abstract

The application of single-cell RNA sequencing in COVID-19 research has greatly improved our understanding of COVID-19 pathogenesis and immunological characteristics. In this commentary, we discuss the current challenges, limitations, and perspectives in harnessing the power of single-cell RNA sequencing to accelerate both basic research and therapeutic development for COVID-19 and other emerging infectious diseases.

## Background

COVID-19 pandemic, caused by the SARS-CoV-2 virus, has become a major threat to global public health. Scientific, medical, pharmaceutical, and biotechnology communities have made great efforts to understand its molecular mechanisms and further develop appropriate therapies. Multiple techniques have been adopted for COVID-19 diagnosis and research, such as real-time reverse transcription-polymerase chain reaction (RT-PCR), antigen tests, enzyme-linked immunosorbent assay (ELISA), and RNA sequencing (RNA-seq). These techniques have been widely used for COVID-19 testing and SARS-CoV-2 variant identification and made significant contributions to COVID-19 screening, diagnosis, and management [1]. However, more advanced techniques are needed for investigating the molecular mechanisms of COVID-19 pathogenesis and immunology.

Single-cell RNA sequencing (scRNA-seq) enables the profiling of transcriptomes from thousands of single cells in each sample, followed by cell-type analysis of gene expression with specific conditions. It has revealed insightful findings in many disease studies. Therefore, it has been quickly applied to investigate SARS-CoV-2 infection and immune response at the cellular and molecular levels since spring 2020. Importantly, scRNA-seq has successfully revealed many characteristics of COVID-19. It has helped investigate the receptors’ expression and infected cells of SARS-CoV-2, measure compositional changes of cell subpopulations, profile the transcriptomes and immune signaling pathways during disease progression, characterize the immune repertoire from adaptive immune response, and others [2, 3]. However, there are still challenges that limit a deeper understanding of COVID-19 pathogenesis, the advancement of vaccine and drug development, as well as development of effective clinical interventions.

## The need and limitation of tissue samples for scRNA-seq

COVID-19 is primarily known as a respiratory syndrome, but compelling lines of evidence have suggested the multiorgan involvement in the disease. When taking tissue samples from COVID-19 patients, strict ethical requirements must be met. This restricts the acquirable tissue samples from patients, limiting a thorough examination of tissues from various organs. Currently, the majority of COVID-19 scRNA-seq studies are limited to peripheral blood mononuclear cells (PBMCs), nasal swabs, and bronchoalveolar lavage fluid (BALF). Collection and profiling of tissue from all representative organs could provide a multi-system landscape view of COVID-19.

Recent attempts to elucidate COVID-19 pathophysiology across the human body are praiseworthy. One good tissue source is from autopsy. Remarkably, single-cell atlases of lung, kidney, liver, and heart autopsy tissue samples from COVID-19 donors were released [4, 5]. While these studies provide important insights into the biological effects across organs during severe SARS-CoV-2 infection, limitations still remain. The samples were collected from critical COVID-19 patients, most likely immunosuppressed. The findings from these patients may not be representative to moderate or severe patients. In addition, the time of tissue sampling in autopsy is much later than the time of infection, disease onset, or progression. As a result, the cell compositions and gene expression profiles of these samples might be significantly different from those in living patients.

Alternative sources of human tissues include in vitro tissue culture and organoids. Three-dimensional organoid models can mimic body tissue structure, and have been successfully applied in complex disease studies. A recent scRNA-seq study using kidney organoids revealed the injury and dedifferentiation of SARS-CoV-2 infected cells, and activation of profibrotic signaling pathways [6]. On the other hand, the cell composition and spatial distribution in organoids derived from induced pluripotent stem cells (iPSCs) do not fully resemble those in the adult organs, and the immune response cannot be modelled without an immune system in the organoid [6]. Therefore, the findings should be carefully interpreted for their implications in vivo. Cross-validation of the results from organoids and autopsy samples is highly recommended to examine the consistency.

The third approach is animal models. Animal experiments can control critical factors like viral exposure dose, duration, and route. Moreover, easy access to the tissues from multiple organs at various time points enables the investigation of spatiotemporal cell atlases, and the tracking of SARS-CoV-2 infection and host immune response dynamics from disease onset to recovery or death. Among animal models, non-human primates like macaques are ideal due to their close phylogenetic relationship with humans [7]. Moreover, rodent models like hamsters and transgenic mice are also widely used due to several advantages such as similar disease phenotype, easy breeding, experimental operation, and low cost [8]. Notably, the susceptibility to SARS-CoV-2 infection varies among species, typically with lower rates in non-human animals than humans.

## The application of scRNA-seq to various COVID-19 conditions

Most COVID-19 scRNA-seq studies aim to find the difference between COVID-19 patients and controls. The emergence of new SARS-CoV-2 variants also calls for further scRNA-seq studies to study differences in their impacts on immunity.

Several variants have become more infectious when viruses keep evolving. Considering that reduced antibody binding affinity and neutralization to variants like B.1.351, B.1.617.2 and B.1.1.529 (including BA.1, BA.2, BA.3, BA.4, BA.5 and descendent lineages) often induce immune escape, there is pressing need to study the adaptive immune response in patients infected by new dominant variants. Since earlier studies only recruited patients infected by previously circulating variants, current and future studies should include the latest dominant variants to reveal their unique immunological features [9]. Inclusion of both vaccinated and unvaccinated patients will further unveil how vaccines affect immune repertoires and multiple immune cell populations in response to new SARS-CoV-2 variants.

Apart from immediate syndrome characteristics, some convalescent individuals report post-acute sequelae, such as dyspnea, fatigue, headache and dysgeusia. Study of the molecular mechanisms of sequelae is an urgent issue. scRNA-seq analysis of PBMCs from patient with post-acute sequelae may reveal symptom-specific immune signatures of different cell populations and help identify sequela-anticipating factors [10]. Even though such sequela studies have similar limitation with tissue samples, scRNA-seq can take full advantage of acquirable samples over consecutive periods of time. The trajectory inference analysis of scRNA-seq data is powerful and unique for longitudinal studies. It can delineate the changes in cell proportions and the shifts in gene expression programs underlying the conditions from acute symptoms to chronic complications.

## Moving towards single-cell multi-omics

In addition to the more popular scRNA-seq, other single-cell technologies have been developed to profile different types of molecules or regulation. Single-cell Assay for Transposase Accessible Chromatin sequencing (scATAC-seq) can profile chromatin accessibility, which suggests the transcription regulatory activity. Ultra-high sensitivity mass spectrometry and Cellular Indexing of Transcriptomes and Epitopes by Sequencing (CITE-seq) can measure protein expression at the single cell level. In addition, the spatial transcriptomics (ST) technology preserves tissue spatial information along with transcriptional profiles. The omics data generated by these technologies complement transcriptomics to provide new perspectives in COVID-19 research. For example, an integrative analysis of multimodal epigenomics, transcriptomics and proteomic data from COVID-19 patients has demonstrated the strength of multi-omics in characterizing the regulatory pathways in immune response [11].

It is worth noting that most COVID-19 scRNA-seq studies have not integrated other omics data yet. Single-cell multi-omics profiling will provide insights into mechanistic modalities like the regulation of immune-related gene expression using scRNA-seq and scATAC-seq, the measurement of SARS-CoV-2 entry receptor expression using CITE-seq, and site-dependent immune responses in tissue using ST.

## Challenges in translating to clinical therapy

scRNA-seq has facilitated the scientific progress in understanding the cellular and molecular mechanisms of COVID-19 pathophysiology and immunology, but there is still a huge gap between its application in basic research and clinical settings such as drug development. Due to inconsistencies in sample collection, insufficient number of subjects, and variation of analytical pipelines, most findings of the immunological landscape have no direct impact on predicting the efficacy of experimental drugs for COVID-19 treatment. Once optimized and standardized, scRNA-seq can assist with monitoring immune responses at the cellular and molecular levels in response to some therapeutic approaches like dexamethasone treatment [12]. As vaccination is currently the most effective method for reducing COVID-19 transmission, scRNA-seq has also been applied to the evaluation of SARS-CoV-2 vaccines like BNT162b2 [13]. In addition, scRNA-seq is also promising for assisting vaccine design and development with its strength in immune profiling at single-cell resolution [14].

Single-cell VDJ sequencing (scVDJ-seq), a technique adapted from scRNA-seq by enriching VDJ sequences in transcripts, can efficiently characterize the heterogeneity in B cell receptor (BCR) and T cell receptor (TCR) repertoires. Compared to bulk RNA-seq and single-cell RT-PCR based techniques, integrative analysis of scRNA-seq and scVDJ-seq data from COVID-19 patients can improve our understanding of the development of human adaptive immune responses to SARS-CoV-2. Transcriptome analysis together with TCR and BCR clonal expansion and lineage analysis will help identify immune components contributing to protection against infection, which can further guide vaccine design and facilitate therapeutic antibody discovery. For example, a study revealed transcriptome signatures and TCR usages of an immunodominant NP_105–113_ specific cytotoxic T cell response, which is associated with mild disease and controls viral replication in vitro. The epitope is promising for future vaccine design [15]. By examining the repertoire of SARS-CoV-2 spike or receptor-binding domain specific B cells from convalescent COVID-19 patients, Scheid et al. identified a group of potent virus-neutralizing antibodies with therapeutic potentials from two clusters of memory B cells and activated B cells, whose frequency correlates with serum neutralizing activity [16]. Many SARS-CoV-2 neutralizing antibodies could be discovered in a short period by scVDJ-seq, which may lead to the rapid development of therapeutic antibodies. However, generation and characterization of thousands of recombinant antibodies to select potent neutralizers after scVDJ-seq is labor-intensive and costly. Thus, we need to build up the knowledge for the efficient selection of neutralizing antibodies through the analysis of scRNA-seq and scVDJ-seq data.

## Concluding remarks

With the rapid application and success of scRNA-seq in COVID-19 research, we scrutinize some major challenges in this field, including the limitation of tissue types, the lack of involvement of new COVID-19 conditions, and the need to translate research into clinical interventions. We summarize the recent endeavor to overcome these challenges, which may provide valuable guidance for future studies. We expect scRNA-seq will be more broadly applied to various COVID-19 studies, especially animals, organoids and antibody discovery. Such knowledge will facilitate better design and develop therapeutic strategies. More importantly, the lessons learned in COVID-19 research prepare us to better harness the power of scRNA-seq in combating future pandemics.

## Data Availability

Not applicable.

## Abbreviations

BALF: Bronchoalveolar lavage fluid
BCR: B cell receptor
CITE-seq: Cellular Indexing of Transcriptomes and Epitopes by Sequencing
iPSC: Induced pluripotent stem cell
PBMC: Peripheral blood mononuclear cell
RT-PCR: Reverse transcription-polymerase chain reaction
scATAC-seq: Single-cell assay for transposase accessible chromatin sequencing
scRNA-seq: Single-cell RNA sequencing
scVDJ-seq: Single-cell VDJ sequencing
ST: Spatial transcriptomics
TCR: T cell receptor
